# Black Sorghum Phenolic Extract Modulates Platelet Activation and Platelet Microparticle Release

**DOI:** 10.3390/nu12061760

**Published:** 2020-06-12

**Authors:** Borkwei Ed Nignpense, Kenneth A Chinkwo, Christopher L Blanchard, Abishek B Santhakumar

**Affiliations:** 1 School of Biomedical Sciences, Charles Sturt University, Locked Bag 588, Wagga Wagga, NSW 2678, Australia; bednignpense@csu.edu.au (B.E.D.); kchinkwo@csu.edu.au (K.A.C.); CBlanchard@csu.edu.au (C.L.B.); 2 Australian Research Council (ARC) Industrial Transformation Training Centre (ITTC) for Functional Grains, Graham Centre for Agricultural Innovation, Charles Sturt University, Wagga Wagga, NSW 2650, Australia

**Keywords:** black sorghum, polyphenols, platelets, platelet microparticles, atherosclerosis

## Abstract

Platelet hyper-activation and platelet microparticles (PMPs) play a key role in the pathogenesis of cardiovascular diseases. Dietary polyphenols are believed to mimic antiplatelet agents by blunting platelet activation receptors via its antioxidant phenomenon. However, there is limited information on the anti-platelet activity of grain-derived polyphenols. The aim of the study is to evaluate the effects of sorghum extract (Shawaya short black 1 variety), an extract previously characterised for its high antioxidant activity and reduction of oxidative stress-related endothelial dysfunction, on platelet aggregation, platelet activation and PMP release. Whole blood samples collected from 18 healthy volunteers were treated with varying non-cytotoxic concentrations of polyphenol-rich black sorghum extract (BSE). Platelet aggregation study utilised 5 µg/mL collagen to target the GPVI pathway of thrombus formation whereas adenine phosphate (ADP) was used to stimulate the P2Y1/P2Y12 pathway of platelet activation assessed by flow cytometry. Procaspase-activating compound 1 (PAC-1) and P-selectin/CD62P were used to evaluate platelet activation- related conformational changes and degranulation respectively. PMPs were isolated from unstimulated platelets and quantified by size distribution and binding to CD42b. BSE treatment significantly reduced both collagen-induced platelet aggregation and circulatory PMP release at 40 µg/mL (*p <* 0.001) when compared to control. However, there was no significant impact of BSE on ADP-induced activation-dependent conformational change and degranulation of platelets. Results of this study suggest that phenolic rich BSE may confer cardio-protection by modulating specific signalling pathways involved in platelet activation and PMP release.

## 1. Introduction

According to a World Health Organisation report, cardiovascular diseases accounted for an estimated 31% of deaths globally with majority being a result of stroke or heart attack [1,2]. In clinical settings, treatment involves blunting the activity of platelets using antiplatelet drugs. These drugs interfere with the thrombotic pathophysiology—wherein a rupture of an atherosclerotic plaque triggers platelet hyper-activation resulting in unwanted clot formation and occlusion of the blood vessel. Macrovesicles referred to as platelet microparticles (PMPs) are released following platelet activation and can contribute to the thrombotic situation [3,4].

The several signalling pathways involved in platelet activation and thrombus formation include receptor-agonist pathways such as P2Y1/P2Y12-ADP, GPVI-collagen, PAR1-thrombin and the COX-1-thromboxane [5]. An agonist such as collagen when exposed by atherosclerotic plaque may activate nearby platelets by binding to their GPVI receptor resulting in complex intracellular signalling that produce a conformational change (indicated by GPIIb/IIIa receptor expression), degranulation (indicated by P-selectin secretion) and subsequent formation of platelet aggregates [5]. In addition, PMP released upon activation possess adhesive and pro-coagulant platelet-derived receptors that further enhance thrombus formation, thereby acting as biomarkers of platelet activation [3]. The common antiplatelet agents, clopidogrel and aspirin, used in clinical treatments inhibit platelet activation and its circulating biomarkers by selectively targeting P2Y1/P2Y12-ADP and COX-1-thromboxane respectively [6]. Unfortunately, because of the unresponsiveness and side effects associated with administration there have been considerable research in dietary bioactive agents known as polyphenols [7].

One such example of a polyphenol-rich functional food is sorghum whole grain. Although mainly used as animal feed, studies have demonstrated that it possesses anti-inflammatory, anti-cancer and antioxidant properties which add value to it as a food for human consumption [8]. Sorghum of different types exist that are classified based on the pigmentation of the pericarp and vary in their phenolic content [9]. The polyphenols found in sorghum that contribute to its bioactivity include flavonoids, hydroxybenzoic acids and hydroxycinnamic acid [8]. Furthermore Francis et al. [10] recently demonstrated that black sorghum rich in catechins and their derivatives may confer cardioprotective properties. The treatment of human umbilical vein cells with flavonoid-rich extract was found to prevent oxidative stress-related endothelial dysfunction through the modulation of gene expression. 

Furthermore, these cardio-protective benefits of polyphenols apply in the context of platelet function. Several studies have demonstrated that polyphenols may inhibit platelet activation, adhesion, degranulation and aggregation by targeting specific thrombogenic pathways for example P2Y_1_/P2Y_12_-ADP, GPVI-collagen, PAR1-thrombin and the COX-1-thromboxane. As reviewed by Ed Nignpense et al. [11] many of the studies that investigate the polyphenol impact on platelet function and PMP generation utilise aggregometry and flow cytometry. However there is limited research on sorghum-derived polyphenols in modulating biomarkers of platelet activation. This study aims to investigate the impact of black sorghum derived polyphenol extracts on collagen-induced platelet aggregation, ADP-induced platelet activation and PMP generation.

## 2. Materials and Methods

### 2.1. Research Ethics

The study protocol was approved by the Charles Sturt University Human Research Ethics Committee (HR17012) and the Institutional Biosafety Committee (19HB02). The study was performed in compliance with relevant laws and institutional guidelines. 

### 2.2. Volunteer Recruitment 

Eighteen healthy volunteers between 18–65 years of age (9 males and 9 females) were recruited from Charles Sturt University and the local community. Informed consent was obtained from all participants prior to commencement of the study. The criteria for recruitment involved normal health status with no history of conditions such as cardiovascular, metabolic, liver or lung disease. Other parameters that could affect the integrity of the analysis such as alcohol consumption, smoking, pregnancy, allergies or venepuncture difficulty were considered during the recruitment process. A health screening questionnaire was used to assess the already mentioned parameters. A dietary questionnaire (adapted from WINTEC and NZ academy of sport) was used to assess the usual dietary intake of volunteers and to avoid recruitment of participants on a high antioxidant diet. The cut-off figure for each type of food listed in the questionnaire was based on nutrient reference ranges for Australia and New Zealand—recommended daily nutrient intake values.

### 2.3. Blood Collection and Processing

After fasting for at least 8 h, whole blood was collected from each participant by a trained phlebotomist into a tri-potassium ethylene diamine tetra-acetic acid (EDTA-1.8 mg/mL concentration) anticoagulant tube (Vacuette Greiner Bio-one, Interpath Services, Heidelberg West, VIC, Australia) and a tri-sodium citrate (28.12 g/L concentration) anticoagulant tube (Becton, Dickson and Company, North Ryde, NSW, Australia). A 20-mL syringe (Becton, Dickson and Company, North Ryde, NSW, Australia) and 21-gauge 1.5-inch needle (Terumo Medical Corporation, Macquarie Park, Australia) were used to draw blood from the median cubital vein. The purpose of choosing a larger gauge was to avoid the activation of platelets while drawing or dispensing blood. Utmost care was taken to ensure samples were not obtained through a traumatic collection and that none contained obvious clots. In addition, care was taken to ensure minimal specimen handling and agitation in order to prevent artefactual platelet activation. The first 2 mL of blood was discarded before drawing into the tri-sodium citrate tubes in order to avoid the risk of collecting platelets activated by venepuncture. Tri-sodium tubes were used for aggregometry and flow cytometry assays whereas the EDTA was used to perform full blood examinations.

### 2.4. Full Blood Examination

Using an Abbott CELL-DYN Emerald 22 Haematology Analyser (Abbott Diagnostics, Illinois, USA), a full blood examination (FBE) was performed on all samples. The FBE results of volunteers indicated that the blood cell parameters were within normal reference ranges as determined by the Royal College of pathologists of Australia. Individuals with cell counts, especially platelet counts, outside of the reference range were excluded from the study. Quality control validation and maintenance were all performed according to the Abbott CELL-DYN Emerald 22 Haematology Analyser manual. 

### 2.5. Extraction of Black Sorghum Polyphenols

Sorghum (*Sorghum bicolor*) samples of six different pericarp varieties were obtained from glasshouse trials conducted by Curtin University, Perth, Western Australia. Six pigmented varieties of sorghum were cultivated under the same conditions, grown in a glasshouse equipped with Lumisol Clear AF cover (200 μm thick, at a transparency of ca UV-A 94%, UV-B 84% and photosynthetic active radiation (PAR, 400-700 nm) 93% [8]. Extraction and analysis of phenolic composition and antioxidant activity were performed previously using methods described by Rao et al. [8]. Among the different sorghum varieties, the black pericarp variety (Shawaya short black 1) was selected for this study because of its relatively high antioxidant activity when analysed with ferric reducing antioxidant power (FRAP; 20.19 ± 2.69 mg/g TE) and 2,2-dipheny-1-picrylhydrazyl (DPPH; 18.04 ± 3.53 mg/g TE) antioxidant assays (Supplementary Figure S1, Tables S1 and S2). The highest level of polyphenols found in the BSE included catechin derivatives, catechins and pentahydroxyflavanone-(3->4)-catechin-7-O- glucoside (Supplementary Table S2). Stock concentrations of BSE (20 mg/mL in 50% DMSO) were diluted in phosphate buffered saline (PBS) to achieve desired concentrations (5 µg/mL, 20 µg/mL and 40 µg/mL) in whole blood. Desired concentrations were selected based on viability studies done by Francis et al. [10].

### 2.6. Whole Blood Platelet Aggregometry

Platelets in whole blood were stimulated for aggregation using 5 µg/mL collagen exogenous platelet agonists (DSKH Australia Pty. Ltd., Hallam, VIC, Australia) to investigate the effect of BSE treatment on the platelet aggregation. Five hundred microliters of citrated whole blood were added to 100 µL of 0.1% DMSO control (Sigma-Aldrich, Castle Hill, NSW, Australia) or BSE stock concentrations (5 µg/mL, 20 µg/mL and 40 µg/mL) and mixed with 400 µL of saline. The sample was then incubated at 37 °C for 20 min. Using a Chrono-log model 700 lumi-aggregometer (DKSH Australia Pty. Ltd., Hallam, VIC, Australia) the samples were analysed by means of electrical impedance (ohms) to determine the amount of platelet aggregation occurring in the sample over a 6-min time period (Supplementary Figure S2).

### 2.7. Flow Cytometry 

#### 2.7.1. Standardisation 

Flow-check fluorospheres were run as quality control for optimal laser alignments. Antibody capture beads (Anti-Mouse Ig, K CompBeads, BD Biosciences, North Ryde, NSW, Australia) were used for single colour compensation controls in order to achieve optimal compensation. Megamix beads (0.1 μm, 0.3 μm, 0.5 μm, 1 μm) from Biocytex, Marseille, France were used as per manufacturer’s instructions to set up an appropriate gating to detecting microparticles. They were run before each PMP analysis. 

#### 2.7.2. Measurement of Platelet Activation-Dependent Conformational Change and Degranulation

The effects of BSE on ADP-induced platelet activation were analysed and interpreted using a Gallios flow cytometer (Beckman Coulter, Inc., Lane Cove NSW, Australia). The protocols were adopted from the method described by Santhakumar et al. [12] with some modifications. Platelet activation and thrombogenic indicators were assessed via activation-dependent platelet monoclonal antibodies (mAbs) purchased from Becton, Dickinson and Company, North Ryde, NSW, Australia. Procaspase activating compound-1 (PAC-1)-fluorescein isothiocyanate-fluorescein isothiocyanate was used to detect platelet activation-related conformational change and P-selectin/CD62P-allophycocyanin highlighted activation dependent degranulation. CD42b-phycoerythrin identified the GPIb-IX-V receptor, a common receptor found on the surface of all platelets, activated and resting included. A decreased expression of mAb exhibits alleviation of thrombogenesis. Within 5 min of collection tri-sodium citrated whole blood was used for assay preparation to avoid artefactual activation of platelets. A volume of 40 μL of blood was incubated with DMSO control or the various BSE concentration for 20 min at 37 °C in the dark. A 10-μL mixture of all three monoclonal antibodies (3.33 μL each of PAC-1, CD62P and CD42b) was added to blood samples and incubated for 20 min at room temperature in the dark. To induce platelet activation, 10 μM ADP (Helena laboratories Pty. Ltd., Mt Waverly, VIC, Australia) was added, and samples were incubated for a further 15 min in the dark at room temperature, after which erythrocytes were lysed (575 μL of 10 % lysing solution). Samples were thoroughly vortexed to ensure homogeneity and incubated in the dark at room temperature for a further 15 min and then analysed. In all, 10,000 platelet events were acquired, gated based on light scatter and CD42b mAb expression and activated platelets were articulated as mean fluorescence intensity (MFI) (Supplementary Figure S3).

#### 2.7.3. Measurement of Circulatory PMPs

Using the microparticle gating established with Megamix beads, PMPs were identified and quantified by size distribution and binding to CD42b (Supplementary Figure S4). The protocol for circulatory PMP analysis was adapted from Lu et al. [13]. A volume of 1 mL whole blood was added to micro-centrifuge tubes in the presence of PGE1 (120 nmol/L; Sigma-Aldrich, Castle Hill, NSW, Australia). PGE1 was added to prevent artefactual activation during centrifugation. The blood samples were incubated with the respective 100 μL BSE concentrations and DMSO control for 20 min at 37 °C in the dark. Each sample was then centrifuged for 15 min at a 1000 rpm and the resultant platelet rich plasma (PRP) was discarded. The remaining blood was spun a further 15 min at 3000 rpm. The supernatant rich in PMPs (40 μL) was collected into flow tubes and incubated with 4 μL of CD42b and 6 μL of stain buffer (Becton, Dickson and Company, North Ryde, NSW, Australia) in dark room for 15 min. Four percent formaldehyde was used to fix any activation of platelets left in the supernatant. After a 10-min incubation period the samples were run for PMP analysis on the flow cytometer.

### 2.8. Statistical Analysis

A two-way ANOVA following Tukey’s post comparison test was performed using GraphPad Prism version 8.0 for Windows (GraphPad Software, La Jolla, California, USA). A minimum sample size of 14 participants in total was required for 80% power to detect a 5% variation in the laboratory parameters measured where a 3–5% standard deviation exists in the population, assuming an alpha error of 0.05. All the data were expressed as mean ± standard deviation (SD). Differences between the groups were significant when *p* < 0.05. Any significant statistical interactions were included in the analysis where applicable.

## 3. Results

The baseline parameters including full blood counts for all 18 participants were within normal reference ranges set by the Royal College of Pathologists of Australasia (Table 1) [14].

### 3.1. Effect of BSE on Whole Blood Platelet Aggregation and Platelet Activation

**Figure 1:**
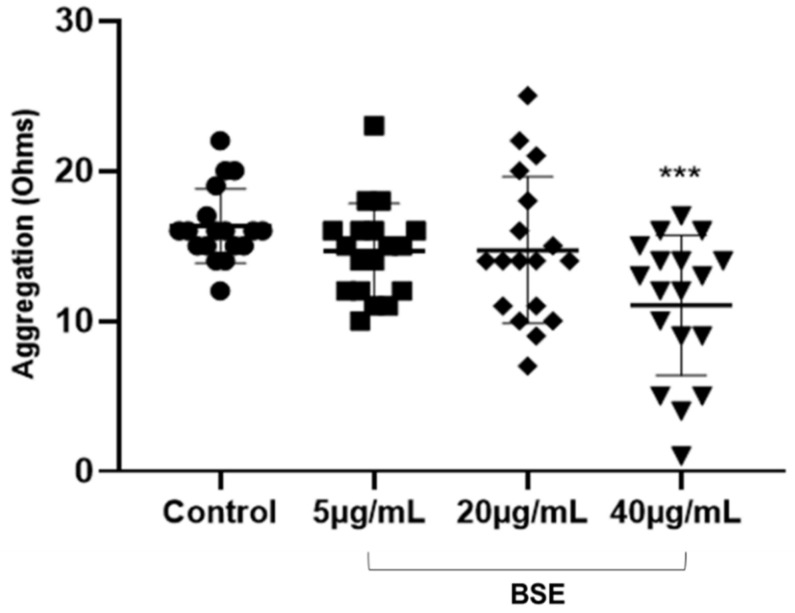
The effect of varying concentrations of BSE on collagen-induced aggregation. BSE at 40 µg/mL significantly reduced platelet aggregation (5.3 ± 1.3; *p* value = 0.0004). BSE at 5 µg/mL and 20 µg/mL did not reduce platelet aggregation when compared to control (*p* value > 0.1). N = 18 and data is represented in aggregation (Ohms) versus BSE concentrations. *** signifies statistical significance *p* < 0.001 compared to control. Error bars expressed as mean ± SD.

BSE at 40 µg/mL concentration significantly reduced platelet aggregation stimulated by collagen by 19 % (*p* = 0.0004) (Figure 1). BSE at lower concentrations did not exhibit any significant reduction in aggregation.

It was observed that whole blood treatment with the varying concentrations of BSE did not significantly affect ADP-induced platelet conformational change and degranulation indicated by PAC-1 and P-selectin expression respectively (Supplementary Figures S5 and S6).

### 3.2. Effect of BSE on Circulatory PMPs 

**Figure 2:**
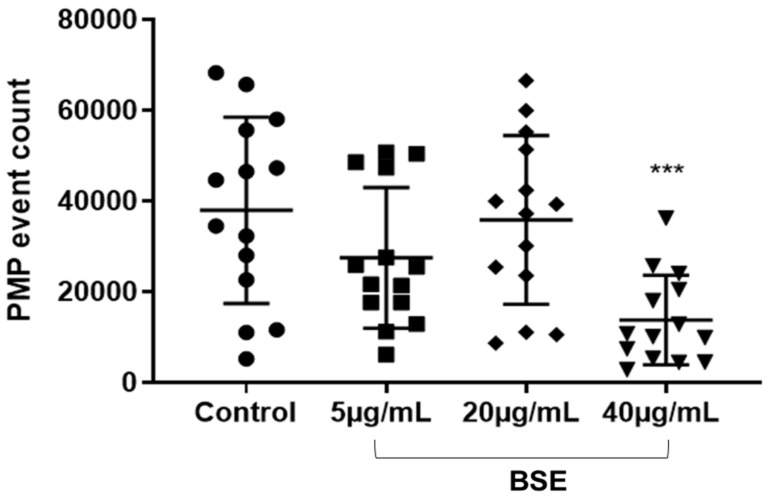
The effect of varying concentrations of BSE on circulatory PMP production in vitro. BSE at 40 µg/mL significantly reduced the amount of circulatory PMPs (<24190 ± 4935, *p* = 0.0008). BSE at 5 µg/mL and 20 µg/mL did not reduce platelet aggregation when compared to control (*p* value > 0.1). N = 14 and data are represented in number of PMP events versus BSE concentrations. *** signifies statistical significance *p* < 0.001 compared to control. Error bars expressed as mean ± SD.

BSE at a concentration of 40 µg/mL significantly reduced the amount of circulatory PMPs in whole blood by 47% (*p* = 0.0008). Lower concentrations of BSE did not exhibit any significant reduction to the amount circulatory PMPs (Figure 2).

## 4. Discussion

There is growing interest in understanding the therapeutic benefits of functional foods. Sorghum for example is one of the functional foods that is showing promise in this area. With sorghum-derived polyphenols already having demonstrated anti-inflammatory, anti-cancer and antioxidant properties, the current study aimed to evaluate the effects of polyphenol-rich BSE on platelet function in terms of aggregation, conformational change, degranulation and circulatory PMP production [8,9,10]. It was observed that BSE significantly inhibited collagen-induced platelet aggregation and decreased the release of circulatory PMPs but did not have a significant effect on ADP-induced platelet conformational change or degranulation. Although these results do not reflect a typical dose-dependent inhibition, they suggest a potential role of BSE polyphenols at optimum concentrations to interfere with pathways in the GPVI-collagen signalling and the release of circulatory PMPs but little or no effect on P2Y_1_/ P2Y_12_-ADP pathway. 

To the best of our knowledge only a few studies have investigated the antiplatelet effects of sorghum extracts. Li, Yu and Fan et al. [15] extracted alditols and monosaccharides from sorghum vinegar to evaluate their anti-aggregation activity using the turbidimetric method. Results from their study indicated a significant dose-dependent inhibition of aggregation via multiple agonists, arachidonic acid, collagen, ADP and thrombin. Furthermore, a different study by Fan et al. [16] reported in vitro inhibition of ADP- and thrombin- induced rabbit platelet aggregation by methanolic extracts of aged sorghum vinegar with the half maximal inhibitory concentrations (IC_50_) of 1.7 ± 0.3 and 8.9 ± 1.9 mg/mL respectively. When rats were orally administered the extracts (>100 mg/kg), both collagen- and epinephrine-induced pulmonary thrombosis were inhibited. In comparison with the present study it is to be noted that these studies employed platelet-rich plasma rather than whole blood hence not accounting for the possible involvement of other blood cells and extracellular mediators involved in thrombus formation. In addition, sorghum vinegar extracts were used at higher concentrations; milligrams compared to micrograms used in this study. This raises the question of bioavailability and the importance of employing physiological concentrations of extracts.

Although the BSE concentration of 40 µg/mL at which antiplatelet effect were observed is relatively lesser in concentration than used in the other studies, the most bioactive compounds with respect to antioxidant activity were catechins and other flavonoids which are usually considered to have low bioavailability [8]. It has been suggested that the total plasma polyphenol concentration rarely exceeds 1 µM and that their antiplatelet effects are only found at high non-physiological concentrations (greater than 50 µM) [17,18]. However, it is likely that these plasma concentrations are underestimations due to the ability of polyphenols to bind to the surface of red blood cells and thereby exert their bioactivity [19]. Furthermore polyphenols (structurally related to catechin) and their metabolites have been shown to inhibit platelet function in vitro [20]. This highlights the possibility of sorghum catechins and their metabolites having antiplatelet effects in vivo despite bioavailability concern. Interestingly, an in vivo human dietary intervention trial compared consumption of red and white wholegrain sorghum-based pasta to a control pasta in order to investigate its acute effect on the total phenol content and antioxidant activity in the plasma of healthy subjects [21]. Results showed that when compared to the control pasta, the red sorghum pasta showed significantly increased net plasma phenolic content and antioxidant activity post consumption (from 216.90 ± 2.62 at baseline to 269.40 ± 2.33 at 2 h; *p* < 0.001), thus demonstrating a plausible correlation between antioxidant activity and sorghum polyphenol consumption—which in turn may contribute to antiplatelet effects. 

The antiplatelet effects was observed with BSE-included inhibition of collagen-induced aggregation and circulatory PMP production but no effect on ADP-induced platelet activation. The absence of antiplatelet effects on the P2Y_1_/ P2Y_12_-ADP activation pathway suggests that BSE polyphenols are not mimicking the action of drugs such as clopidogrel that antagonise P2Y_12_ receptor activation [22]. However, the inhibition of collagen-induced aggregation suggest that BSE polyphenols interfere with GPVI-collagen signalling pathways by either blunting the GPVI receptor directly or by other mechanisms [5]. Previous studies have demonstrated that flavonoids, specifically quercetin and catechin, can act synergistically to inhibit collagen-induced aggregation by blunting the associated burst of H_2_O_2_ and subsequent PLC activation [23,24]. Thus, a possible mechanism of inhibition BSE flavonoids may be a synergistic antagonism of the positive feedback activation of intracellular signals triggered by H_2_O_2_. Moreover, it has been shown that the phosphorylation cascade initiated by collagen can be inhibited by flavonoids [25,26]. Flavones, especially apigenin and luteolin, by virtue of a double bond in the C2-C3 and the keto group in C4 can also inhibit collagen-induced activation by antagonizing the TxA_2_ receptor activation which is also involved in the positive feedback loop [27]. Besides inhibition of the GPVI-collagen signalling, BSE polyphenols showed inhibition of the circulatory PMP production. 

To the best of our knowledge, this is the first study investigating the effect of sorghum-derived polyphenols on PMP production. In contrast to this study, other PMP studies have employed the use of Annexin V as well as the platelet specific antibody CD42b, to identify pro-coagulant PMPs by their phosphatidylserine expression and to limit background noise [28]. However, because of the heterogeneity of PMPs, not all PMPs express phosphatidylserine [29]. Moreover, the measurement of CD42b-positive PMPs alone is significant as its increase has been associated with an increased risk of coronary heart disease [30]. From the current study, the significant inhibition of CD42b-positive circulatory PMPs observed in vitro may be attributed to the antioxidant properties of BSE polyphenols. It is believed that the inhibition of PMP generation may be the result of neutralisation of H_2_O_2_, scavenging of other free radicals or interaction with intracellular signalling leading to PMP release. 

The juxtaposition of both the present study and that of an earlier study by Francis et al. [10] highlights the multifaceted role of BSE polyphenols in cardio-protection. The group investigated the effects of BSE polyphenols on the expression of antioxidant- and inflammatory-linked genes involved in endothelial dysfunction under oxidative stress. Results indicated that BSE polyphenols alleviate oxidative stress–induced damage to endothelial cells. Since vascular dysfunction is a precursor to cardiovascular diseases, the current study builds upon earlier findings by exhibiting the antiplatelet effects of BSE. In the context of endothelial dysfunction, platelet activation and circulatory PMPs play central roles in the pathogenesis of atherothrombosis. The disruption of the plaque exposes collagen that binds to the GPVI receptor resulting in platelet activation and subsequent thrombus formation [31]. Circulatory PMPs may contribute to thrombosis via GPIb-IX-V receptor binding and have a pro-inflammatory effect to promote the development of the plaque [32]. Therefore, by reducing collagen-induced platelet aggregation and circulatory PMP generation, BSE polyphenols may be displaying the potential to augment thrombosis.

## 5. Conclusion and Future Considerations

In summary, the present study contributes to the growing body of literature on bioactivity of sorghum polyphenols and highlights possible mechanisms of antiplatelet action that may result in cardiovascular health benefits. Because of the ability to reduce collagen-induced platelet activation and circulatory PMP generation, BSE polyphenols demonstrate the potential to interfere with pathological processes involved in vascular disorders and thrombotic complications. However, a larger panel of agonist for the flow cytometry and aggregometry studies will aid to further elucidate antiplatelet mechanisms. Because of the bioavailability concerns, well-controlled dietary intervention trials using larger sample sizes to evaluate the antiplatelet effects of sorghum consumption in healthy and pro-thrombotic populations are warranted to justify our findings. Because of the varied phenolic profiles of the different sorghum varieties, further research comparing the antiplatelet therapeutic potential of different grains is also warranted. Furthermore, this study attest to the measurement of circulatory PMPs as a biomarker of platelet activation to assess the bioactivity of functional foods. 

## Figures and Tables

**Table 1:** Baseline full blood counts of participants.

Parameters	Mean ± SD
Age (years)	26 ± 8
WBC (× 10^9^/L)	5.5 ± 1.3
Neutrophil (%)	48.6 ± 9.5
Lymphocytes (%)	37.8 ± 9.2
Monocytes (%)	10.6 ± 2.3
Eosinophils (%)	2.8 ± 1.3
Basophils (%)	0.1 ± 0.1
RBC (× 10^12^/L)	4.6 ± 0.5
Haemoglobin (g/L)	147.9 ± 16.1
PCV (%)	0.41 ± 0.04
MCV (fL)	90.0 ± 3.5
MCH (pg)	35.8 ± 15.2
MCHC (g/dL)	360.1 ± 6.0
RDW (%)	14.9 ± 0.8
Platelet count (× 10^9^/L)	248.3 ± 50.0
MPV	8.41 ± 0.89

Values are represented as mean ± Standard deviation (SD). RBC, red blood cell, PCV, packed cell volume, MCV, mean cell volume, MCH, mean cell haemoglobin, MCHC, mean cell haemoglobin concentration, RDW, red cell distribution width, MPV, mean platelet volume.

## Supplementary Materials

The following are available online at https://www.mdpi.com/2072-6643/12/6/1760/s1, Figure S1: Characterisation of phenolic compounds in BSE. Ultra-high-performance liquid chromatography (UHPLC) was employed to quantify the different phenolic compounds identified by the peak (on top). An online 2,2′-azino-bis(3-ethylbenzothiazoline-6-sulfonic acid) (ABTS) was coupled with UHPLC to quantify the relative antioxidant activity (peaks below) of each compound identified, Figure S2: A report derived from the collagen induced platelet aggregation study using the Chrono-log model 700 lumi-aggregometer (DKSH Australia Pty. Ltd, Hallam, VIC, Australia). The blue tracing represents the control (whole blood with no BSE) and the black tracing represents the whole blood pre-treated with 5 µg/mL BSE. The addition of BSE reduced the maximum platelet aggregation expressed in Ohms from 15 ohms to 11 ohms, Figure S3: A report from the ADP-induced platelet activation analysis using Kaluza Flow Cytometry Software (Beckman Coulter, Brea, CA, USA). Results indicate the gating of whole platelet population (CD42b positive events) and the proportion of activated platelets indicated by PAC-1 and P-selectin expression, Figure S4: A report from the PMP analysis using Kaluza Flow Cytometry Software (Beckman Coulter, Brea, CA, USA). Microparticle gating was established using Megamix beads of standard sizes. PMPs were distinguished from other microparticles by size (0.5 μm - 0.9 μm) and expression of CD42b. The number of CD42b positive events in the microparticle gate was used to quantify the PMPs, Figure S5: The effect of varying concentrations of BSE on PAC-1 expression. BSE did not significantly reduce ADP-induced platelet conformational change detected by PAC-1 expression (*p* values > 0.1 compared to control) N=14 and data is represented in mean fluorescence intensity (MFI) versus BSE concentrations. Error bars expressed as mean ± SD, Figure S6: The effect of varying concentrations of BSE on P-selectin expression. BSE did not significantly reduce ADP-induced platelet degranulation detected by P-selectin expression (*p* values > 0.1 compared to control ) N=14 and data is represented in mean fluorescence intensity (MFI) versus BSE concentrations Error bars expressed as mean ± SD, Table S1: Phenolic composition and antioxidant activity of sorghum varieties on as is basis, Table S2: List of top ten phenolic compounds identified in the black sorghum phenolic rich extracts by Q-TOF LC/MS and quantified using UHPLC-Online ABTS system (Adapted from Rao et al., 2018).

## Abbreviations

ADP	Adenosine diphosphate
ATP	Adenosine triphosphate
BSE	Black sorghum extract
COX-1	Cyclooxygenase-1
DMSO	Dimethyl sulfoxide
EDTA	Ethylene diamine tetra-acetic acid
FBE	Full blood examination
GAE	Gallic acid equivalents
MCH	Mean cell haemoglobin
MCHC	Mean cell haemoglobin concentration
MCV	Mean cell haemoglobin
MFI	Mean fluorescence intensity
MPV	Mean platelet volume
PAR	Protease-activated receptor
PAC-1	Procaspase activating compound-1
PBS	Phosphate buffered saline
PCV	Packed cell volume
PGE1	Prostaglandin E1
PMP	Platelet microparticle
RBC	Red blood cell
SD	Standard deviation
WBC	White blood cell
