# Supplementary material for: Black Sorghum Phenolic Extract Modulates Platelet Activation and Platelet Microparticle Release
Source: Nutrients. 2020 Jun 12;12(6):1760. doi: 10.3390/nu12061760 (PMC7353362; doi:10.3390/nu12061760)
Supplement: Supplementary file 1 [file nutrients-12-01760-s001.pdf]

## Supplementary Materials

Table 1, Figure 1 and Table 2 are the summary of key findings from Rao, S., A.B. Santhakumar, K.A. Chinkwo, G. Wu, S.K. Johnson, and C.L. Blanchard, J. Cereal Sci., 2018, 84, 103.

**Table 1.** Phenolic composition and antioxidant activity of sorghum varieties on as is basis.

| Variety | TPC mg/g GAE | TPAC mg/g CE | DPPH mg/g TE | FRAP mg/g TE |
|---|---|---|---|---|
| Shawaya short black 1 | 11.50 ± 1.81a | 3.02 ± 0.72a | 18.04 ± 3.53a | 20.92 ± 2.69a |
| IS 13116 (Brown) | 3.58 ± 1.63b | 5.55 ± 0.40b | 21.02 ± 5.17a | 4.62 ± 3.19b |
| QL33/QL36 (red) | 0.88 ± 0.17c | 0.47 ± 0.23c | 1.17 ± 0.48b | 4.83 ± 2.54b |
| B923296 (red) | 0.66 ± 0.27c | 0.41 ± 0.28c | 0.41 ± 0.14b | 2.72 ± 1.35b |
| QL12 (white) | 0.24 ± 0.03c | 0.09 ± 0.26c | 0.33 ± 0.10b | 2.31 ± 1.55b |
| QL33 (red) | 0.75 ± 0.23c | 0.60 ± 0.39c | 0.76 ± 0.18b | 3.43 ± 2.37b |

Table 1 presents a comparison of the six pigmented sorghum varieties in terms of their total phenolic content (TPC) Total proanthocyanidin content (TPAC) and their relative antioxidant capacity. 2,2-diphenyl-1-picryl-hydrazyl-hydrate (DPPH) and ferric reducing antioxidant power (FRAP) assays were used to determine antioxidant capacity. Shawaya short black 1 (BSE) showed the highest TPC and antioxidant activity indicated by FRAP.

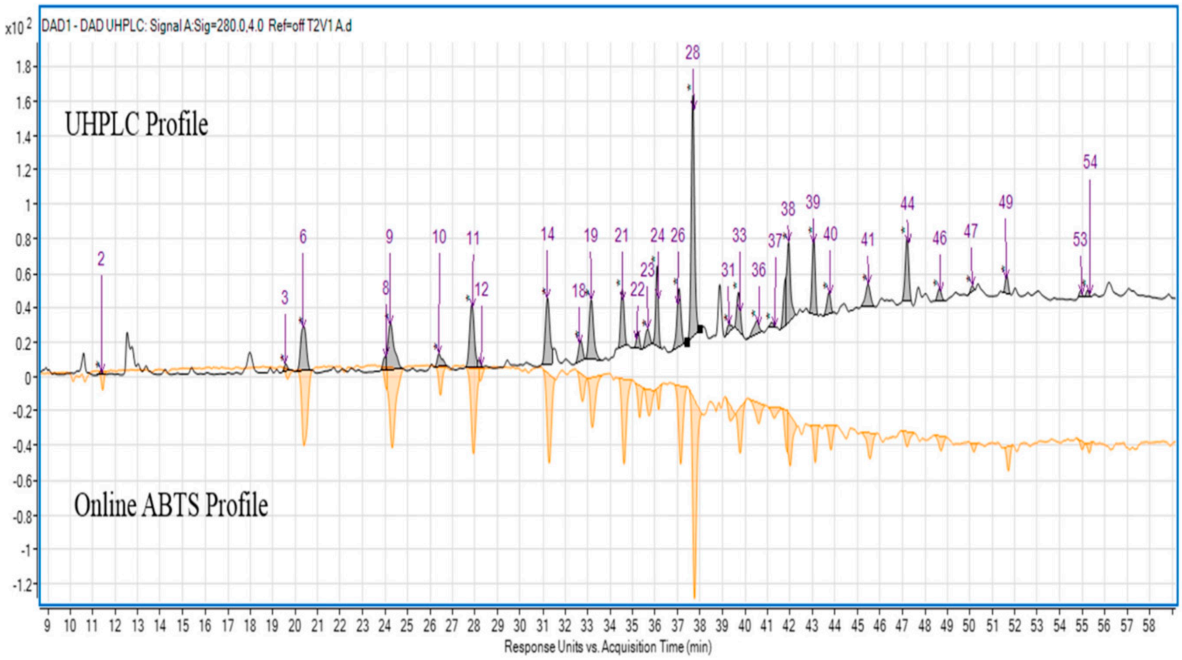


**Figure 1.** Characterisation of phenolic compounds in BSE. Ultra-high-performance liquid chromatography (UHPLC) was employed to quantify the different phenolic compounds identified by the peak (on top). An online 2,2′-azino-bis(3-ethylbenzothiazoline-6-sulfonic acid) (ABTS) was coupled with UHPLC to quantify the relative antioxidant activity (peaks below) of each compound identified.

**Table 2**. List of top ten phenolic compounds identified in the black sorghum phenolic rich extracts by Q-TOF LC/MS and quantified using UHPLC-Online ABTS system (Adapted from Rao et al., 2018)

| Compound | Phenolic quantity (mg 100mg-1 GAE) | Antioxidant activity (mg 100mg-1 TE) |
|---|---|---|
| **Catechin derivative** | 2.11±0.47 | 1.51±0.27 |
| **Catechin** | 1.97±0.36 | 1.54 ± 0.25 |
| **Pentahydroxyflavanone-(3->4)-catechin-7-O- glucoside** | 1.8±0.28 | 2.62 ± 0.36 |
| **Catechin derivative** | 0.66±0.13 | 1.42 ± 0.24 |
| **Pyrano-eriodictyol-(3->4)-catechin-7-Oglucoside** | 0.65±0.14 | 0.78±0.16 |
| **I-O-Caffeoylglycerol-O-glucoside** | 0.65±0.12 | 1.74±0.33 |
| **N'.n'-dicafferoylspermidine** | 0.61±0.12 | 0.98±0.15 |
| **Pyrano-eriodictyol-(3->4)-catechin-7-O-glucoside isomer** | 0.55 ± 0.08 | 0.60 ± 0.06 |
| **Robinetinidol-(4alpha->6)-catechin-(6>4alpha)-robinetinidol** | 0.53±0.11 | 1.25 ± 0.20 |
| **Pyrano-naringenin-(3->4)-catechin-7- Oglucoside isomer** | 0.53±0.07 | 0.42 ± 0.06 |

From Figure 1 the peaks with the highest phenolic content and relative antioxidant activity are shown here. The phenolic content of the identified peaks from Figure 1 were quantified using a gallic acid standard curve data expressed as mg $g^{-1}$ gallic acid equivalents (GAE). Trolox was used to quantify the ABTS radical scavenging activity and was expressed as mg 100 $g^{-1}$ Trolox equivalents (TE).

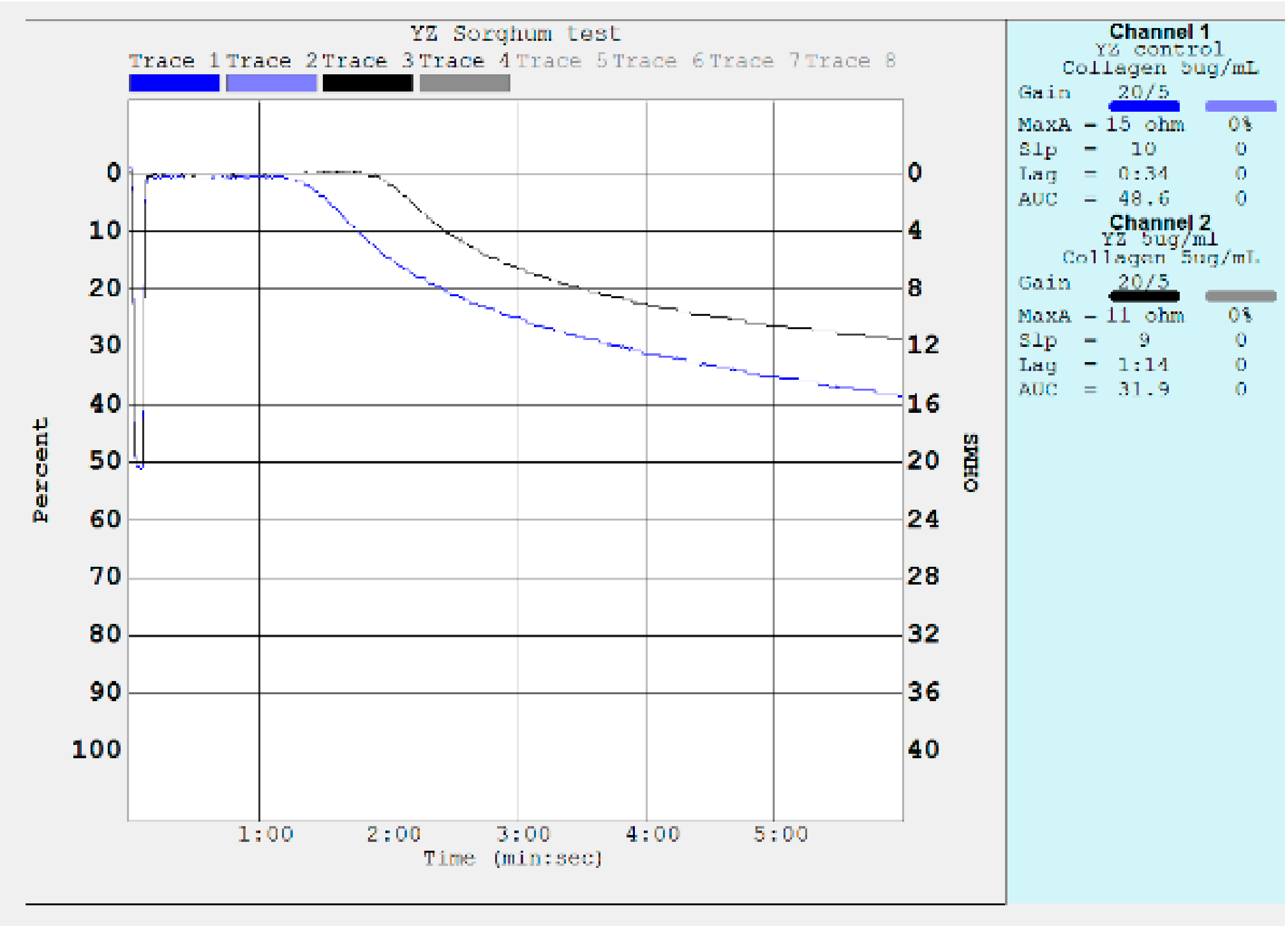


**Figure 2.** A report derived from the collagen induced platelet aggregation study using the Chrono-log model 700 lumi-aggregometer (DKSH Australia Pty. Ltd, Hallam, VIC, Australia). The blue tracing represents the control (whole blood with no BSE) and the black tracing represents the whole blood pre-treated with 5 µg/mL BSE. The addition of BSE reduced the maximum platelet aggregation expressed in Ohms from 15 ohms to 11 ohms.

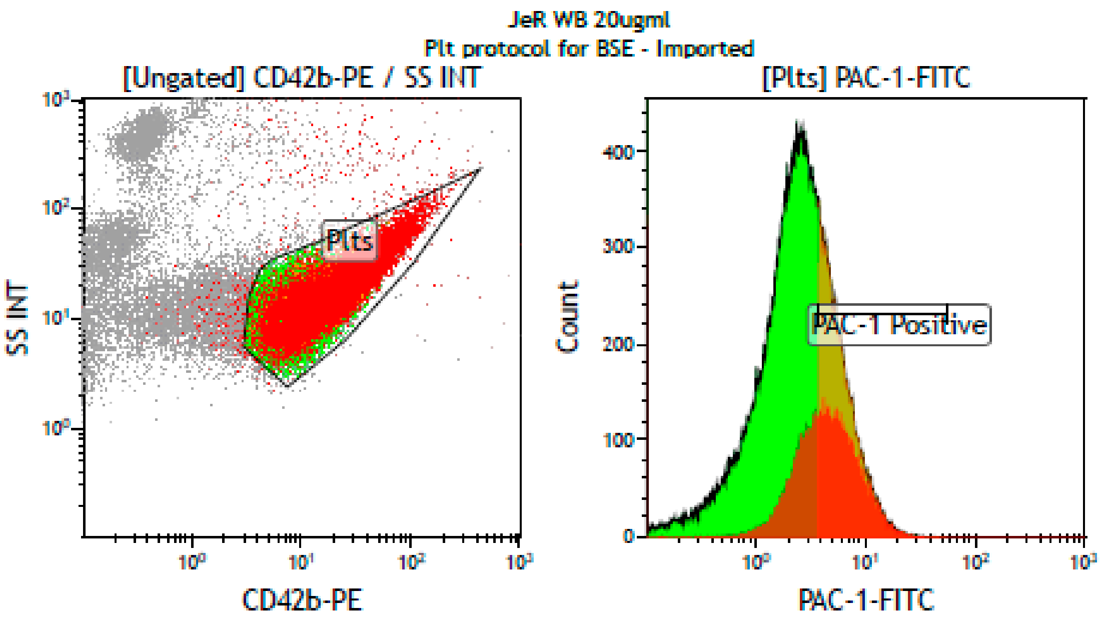


| Gate | Number | %Gated | X-AMean |
|---|---|---|---|
| All | 85,028 | 100.00 | 3.17 |
| PAC-1 Positive | 24,934 | 29.32 | 6.41 |

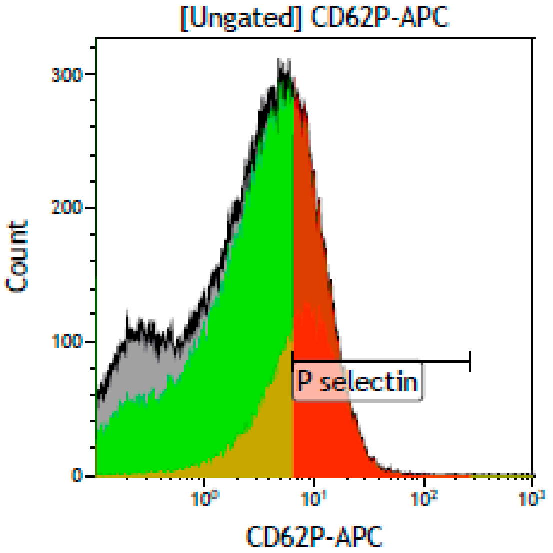


| Gate | Number | %Gated | X-AMean |
|---|---|---|---|
| All | 100,000 | 100.00 | 4.80 |
| P selectin | 24,705 | 24.71 | 12.34 |

**Figure 3.** A report from the ADP-induced platelet activation analysis using Kaluza Flow Cytometry Software (Beckman Coulter, Brea, CA, USA). Results indicate the gating of whole platelet population (CD42b positive events) and the proportion of activated platelets indicated by PAC-1 and P-selectin expression.

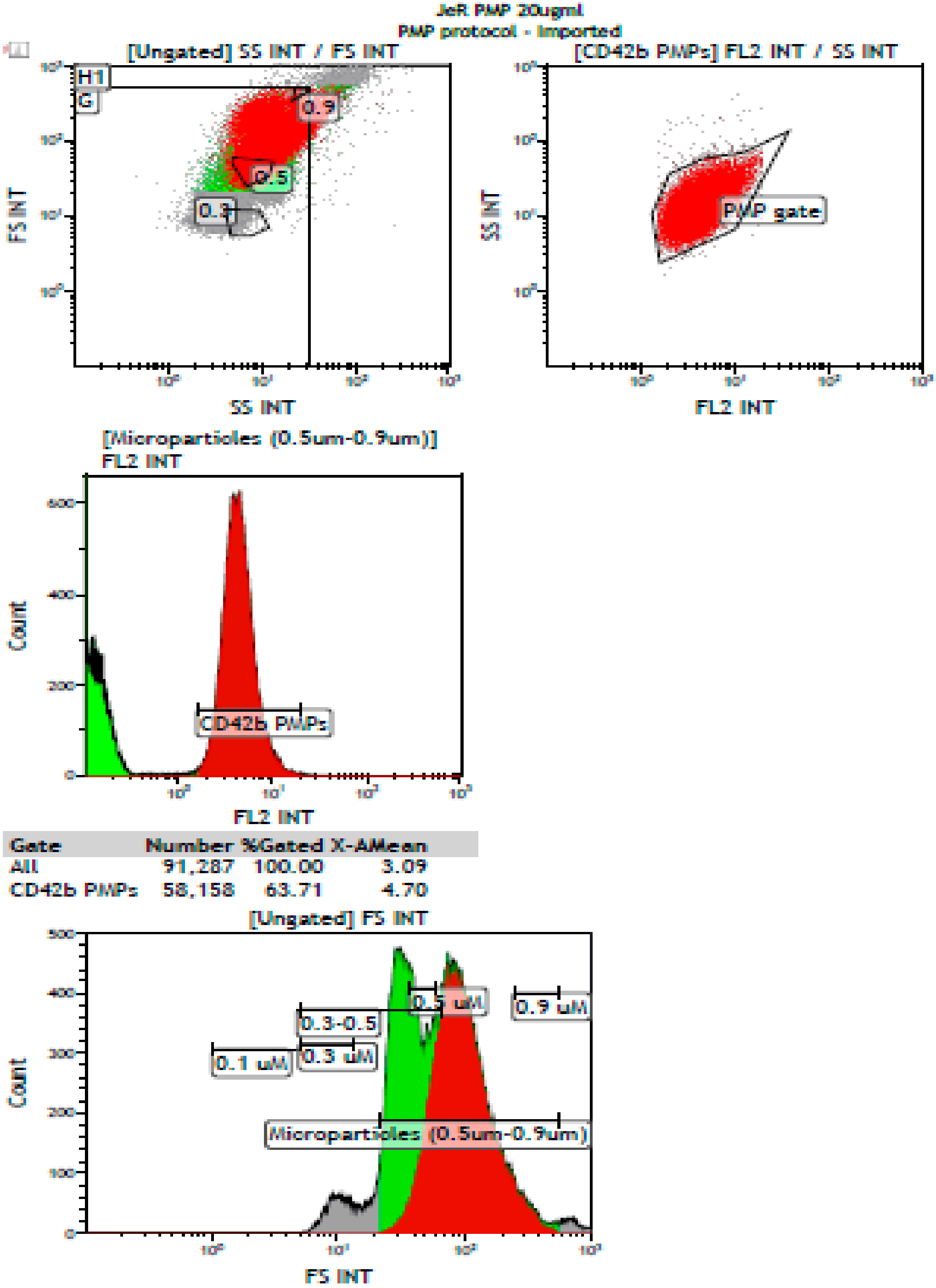


**Figure 4.** A report from the PMP analysis using Kaluza Flow Cytometry Software (Beckman Coulter, Brea, CA, USA). Microparticle gating was established using Megamix beads of standard sizes. PMPs were distinguished from other microparticles by size (0.5 µm - 0.9 µm) and expression of CD42b. The number of CD42b positive events in the microparticle gate was used to quantify the PMPs.

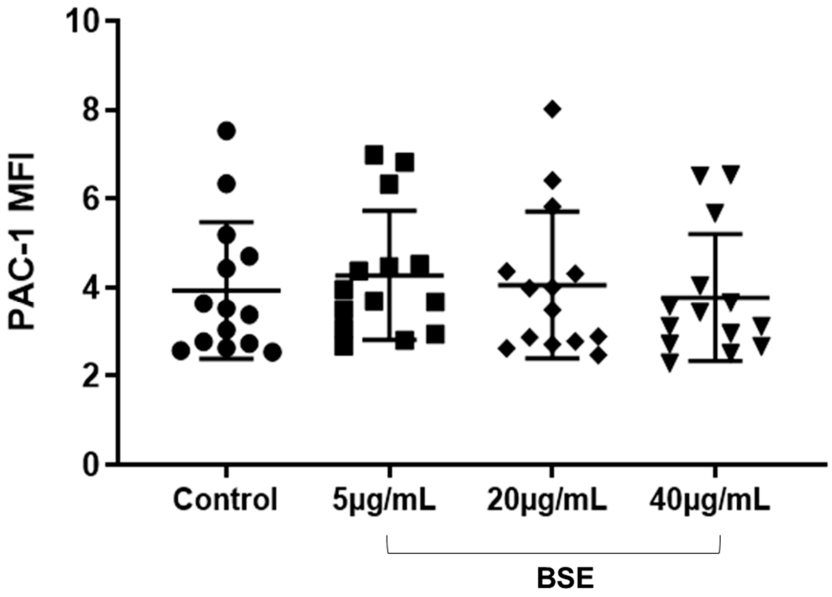


**Figure 5.** The effect of varying concentrations of BSE on PAC-1 expression. BSE did not significantly reduce ADP-induced platelet conformational change detected by PAC-1 expression (*p* values > 0.1 compared to control) N=14 and data is represented in mean fluorescence intensity (MFI) versus BSE concentrations. Error bars expressed as mean ± SD.

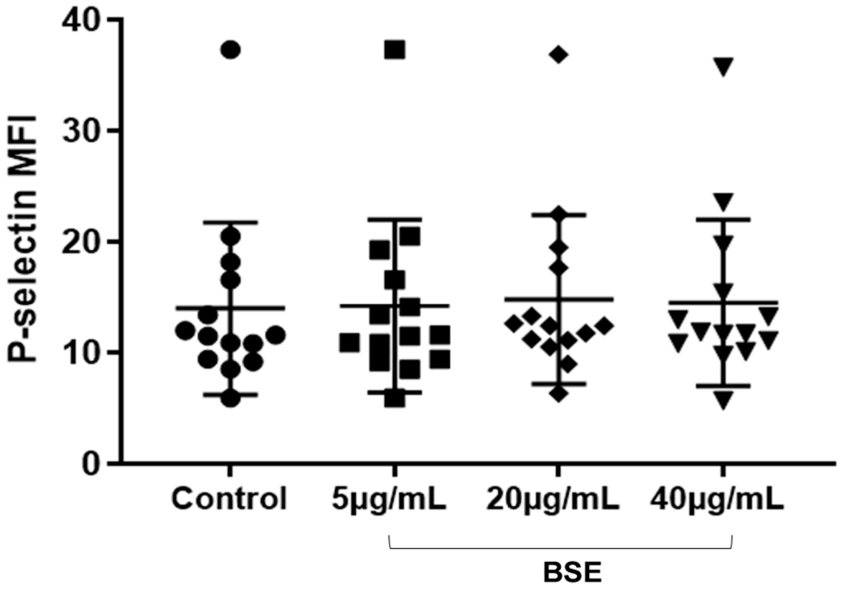


**Figure 6.** The effect of varying concentrations of BSE on P-selectin expression. BSE did not significantly reduce ADP-induced platelet degranulation detected by P-selectin expression (*p* values > 0.1 compared to control ) N=14 and data is represented in mean fluorescence intensity (MFI) versus BSE concentrations Error bars expressed as mean ± SD.
